# How providers influence the implementation of provider-initiated HIV testing and counseling in Botswana: a qualitative study

**DOI:** 10.1186/s13012-015-0361-7

**Published:** 2016-02-11

**Authors:** Shahira Ahmed, Till Bärnighausen, Norman Daniels, Richard Marlink, Marc J. Roberts

**Affiliations:** 1 Harvard T. H. Chan School of Public Health, 677 Huntington Avenue, Boston, MA 02115 USA; 2 Africa Centre for Population Health, University of KwaZulu-Natal, KwaZulu-Natal, South Africa

**Keywords:** HIV, Provider-initiated testing and counseling, Human resources, Implementation, Health systems, Provider behavior, Qualitative research

## Abstract

**Background:**

Understanding the motivations and perspectives of providers in following guidance and evidence-based policies can contribute to the evidence on how to better implement and deliver care, particularly in resource-constrained settings. This study explored how providers’ attitudes and behaviors influenced the implementation of an intervention, provider-initiated HIV testing and counseling, in primary health care settings in Botswana.

**Methods:**

Using a grounded-theory approach, we purposively selected and interviewed 45 providers in 15 facilities in 3 districts and inductively analyzed data for themes and patterns.

**Results:**

We found that nurses across facilities and districts were largely resistant to offering and delivering provider-initiated testing and counseling for HIV (PITC) for three reasons: (1) they felt they were overworked and had no time, (2) they felt it was not their job, and (3) they were afraid to counsel patients, particularly fearing a positive HIV test. These factors were largely related to health system constraints that affected the capacity of providers to do their job. An important underlying themes emerged: nurses and lay counselors were unsatisfied with pay and career prospects, which made them unmotivated to work in general. Variations were seen by urban and rural areas: nurses in urban areas felt generally overworked and PITC was seen as contributing to the workload. While nurses in rural areas did not feel overworked, they felt that PITC was not their job and they were unmotivated because of general unhappiness with their rural posts.

**Conclusions:**

The attitudes and behaviors of providers and barriers they faced played a critical role in whether and how PITC was being implemented in Botswana. Provider factors should be considered in the improvement of existing PITC programs and design of new ones. Addressing constraints faced by providers can do more to improve supply of human resources than merely recruiting more providers.

## Background

Strengthening health systems at the facility level and the successful implementation of interventions require an effective workforce at the frontlines [1, 2]. Recruiting sufficient numbers of providers to deliver care does not automatically translate to efficient or effective implementation of standards of care and guidelines. Understanding the motivations and perspectives of providers in following guidance and evidence-based policies can contribute to the evidence on how to better implement and deliver care, particularly in resource-constrained settings. This study explored how providers’ attitudes and behaviors influenced the implementation of an intervention, provider-initiated HIV testing and counseling, in primary health care settings in Botswana.

Global and national guidance uphold provider-initiated testing and counseling (PITC) as an important strategy in high HIV prevalence areas for diagnosing HIV cases in clinical settings and connecting patients to treatment and care [3, 4]. While it has been shown to be an effective strategy, barriers arise in moving from studies and pilot programs to real settings [5]. A few studies have examined the challenges in implementing PITC [6–8]. One small-scale study in Kenya held focus groups with nurses in outpatient and inpatient settings and concluded that nurses found implementing PITC difficult and stressful particularly in maintaining consent, confidentiality, and counseling [6]. Another study in South Africa addressed factors that played a role in the implementation of PITC in tuberculosis (TB) clinics in the Eastern Cape Province [8]. They found that lack of staff and high workloads, a conflict between the requirements of testing and counseling and available resources, and ambivalence to when and how testing and counseling should be done were all barriers to PITC implementation. Filler et al. [7] assessed forty-five PEPFAR sites in Botswana, Ethiopia, Nigeria, Uganda, and Vietnam and determined that human resources at all sites were stretched too thin. What is less understood is how and why providers’ attitudes and behaviors change the delivery and implementation of PITC, which is the focus of this study.

Botswana was an ideal place to examine the implementation of PITC in primary health care settings. It is a middle-income country in Southern Africa with a population of a little over 2 million where HIV remains the dominant health problem [9]. HIV prevalence was 19 %, and the incidence rate was 2.9 % in the general population in 2013 [10]. In 2002, Botswana was the first African country to establish a free and universal national HIV/AIDS antiretroviral treatment (ART) program, and in 2004, became the first country to introduce PITC in all its public-sector facilities [11]. HIV testing and counseling (HTC) services in Botswana are provided through a network of private, public, and non-governmental organizations and guided by comprehensive national guidance that encompasses both client-initiated and provider-initiated models of HTC [9]. The guidance calls for PITC to be offered routinely by providers to all patients as part of the standard of care regardless of their reasons for presentation [12].

## Methods

### Setting and sampling

The setting was public-sector health facilities in three districts in Botswana. Purposive sampling was used to select health facilities for the study. There are 636 public-sector health facilities and 32 non-governmental organizations delivering HTC in the country [9]. All public-sector facilities were grouped and categorized based on whether they were in urban, rural, or urban village districts; whether they provided ART services or not; and whether they were outpatient clinics, primary health care clinics, or health posts. A total of 15 facilities were selected using these criteria to be in the final sample. Nurses and lay counselors at outpatient clinics were asked to be interviewed at the selected facilities, and informed consent was sought from each participant. Permission for the study was obtained from institutional review boards at the Harvard T.H. Chan School of Public Health and the Health Research and Development Division, Botswana Ministry of Health.

### Data collection

A semi-structured interview guide was constructed and piloted by interviewing three health care professionals and three doctors in facilities not selected for the study. Providers were visited at the facilities after seeking formal permission at the national and district levels. Since visits were conducted during working hours, an appointment was made with the interviewee to return another day if they were too busy. To increase validity, great lengths were taken to build trust with interviewees. Permission was sought verbally for conducting and recording the interviews, and the interview was conducted one-on-one in a private room or office within the facility. No officials, local or national, accompanied the researcher to the sites. The interview began with an introduction of the objectives of the study assuring participants about their anonymity and the confidentiality of the information they shared. This was followed by a set of questions on how PITC was delivered at their facility and then questions on the interviewee’s perceptions on what they thought worked or did not work well in relation to PITC implementation. A discussion then followed on what they found rewarding and challenging about their job in general, and any other issues that the interviewees wanted to raise. The interviews lasted between 30 and 60 min. Forty-five interviews were conducted in total.

### Data analysis

A grounded-theory approach was used in data analyses. Grounded theory is used in qualitative studies to generate theories or hypotheses that may help explain certain practices or that can provide a framework for further research [13]. As described by Corbin and Strauss, a positivist approach to grounded-theory analyses applies a structured and methodical procedure to the analysis, while a constructivist approach allows for a more fluid form of analysis [13]. A positivist approach is more suited and more commonly used in the health and medical sciences and was used here.

The Atlas.ti software was used for data management and analyses. An initial list of broad categories to be used for coding was compiled based on relevant topic areas reflective of the interview questions and the existing literature on PITC. The categories included the type of patient population seen at the clinics, providers’ duties and responsibilities in delivering PITC, the different steps and components necessary for the delivery of PITC, and barriers to PITC implementation. The aim was to capture providers’ views and attitudes in each of these broad categories. This was done to ensure these initial analytic categories had a relationship with the research question and were not arbitrarily selected [8]. Data was transcribed and coded using sentences as the units of analysis. The units of data were then sorted into the pre-selected categories through a process of “open coding.” Observations within each category were labeled with subjective sub-codes that were reflective of the view of the providers under each category, thus following grounded-theory approach to “ground” findings in the data [13]. The sub-codes in each category were reviewed to determine their density and emerging patterns. Berg writes that “a common rule of thumb is that a minimum of three occurrences of something can be considered a pattern” [14]. Once identified, these thematic patterns were further analyzed to develop a matrix that can be used to hypothesize about how and why providers’ attitudes affected delivery of PITC within and across facilities and districts.

## Results

From the total of 45 providers interviewed, 8 were lay counselors and 37 were nurses with varying titles and levels of experience (Table 1). Of the 37 nurses, approximately a quarter were also trained as midwives, and the majority had received training in PITC, with similar distributions across rural, urban, and urban village sites. In this section, findings from the first and second phase of coding are presented first. The views and attitudes of providers were coded under the following broad categories: duties, incentives, PITC components, space, and supplies.

**Table 1:** Characteristics of the providers interviewed in the study (*N* = 45)

	Number	Urban	Rural	Urban village
*N*	45	21	13	11
Nurses	37	17	11	9
Lay counselors	8	4	2	2
Nurses trained as midwives	11	28 %	23 %	18 %
Trained in RHT	28	62 %	62 %	64 %
Average age in years (min, max)	38 (22, 71)	40 (27, 58)	35 (22, 43)	40 (25, 71)
Average years of experience (min, max)	12 (0, 37)	13 (1, 36)	10 (1, 18)	12 (1, 37)
Average years at current facility (min, max)		1 (1, 5)	3 (1, 8)	3 (1, 8)

Within the broad category of “duties,” sub-codes were grouped into what providers thought took up their time or the tasks they found were challenging. The most frequently mentioned sub-codes were tasks related to transporting and coordinating blood samples and patients. Other sub-codes that had more than five mentions included the following: spending time on tasks they were over-qualified for, home follow-up care, completing registers and reports, seeing a large volume of patients, and having one lay counselor on staff. The comment about time spent on transportation and completing registers was largely made by nurses in urban areas. Home visits were most frequently identified by rural providers as taking up the most time.

Within the “incentives” category, data was coded into sub-codes related to aspects providers liked or did not like about their job in general. A few patterns emerged from this sorting. Having an unresponsive or unsupportive supervisor emerged as the most frequent statement made by providers in relation to their job. Other sub-codes that had more than five responses included the following: providers being at their current job only because they cannot find another one, lack of incentive even if non-monetary, lack of confidence in their skills and training, and lack of progress and low pay.

**Fig. 1:**
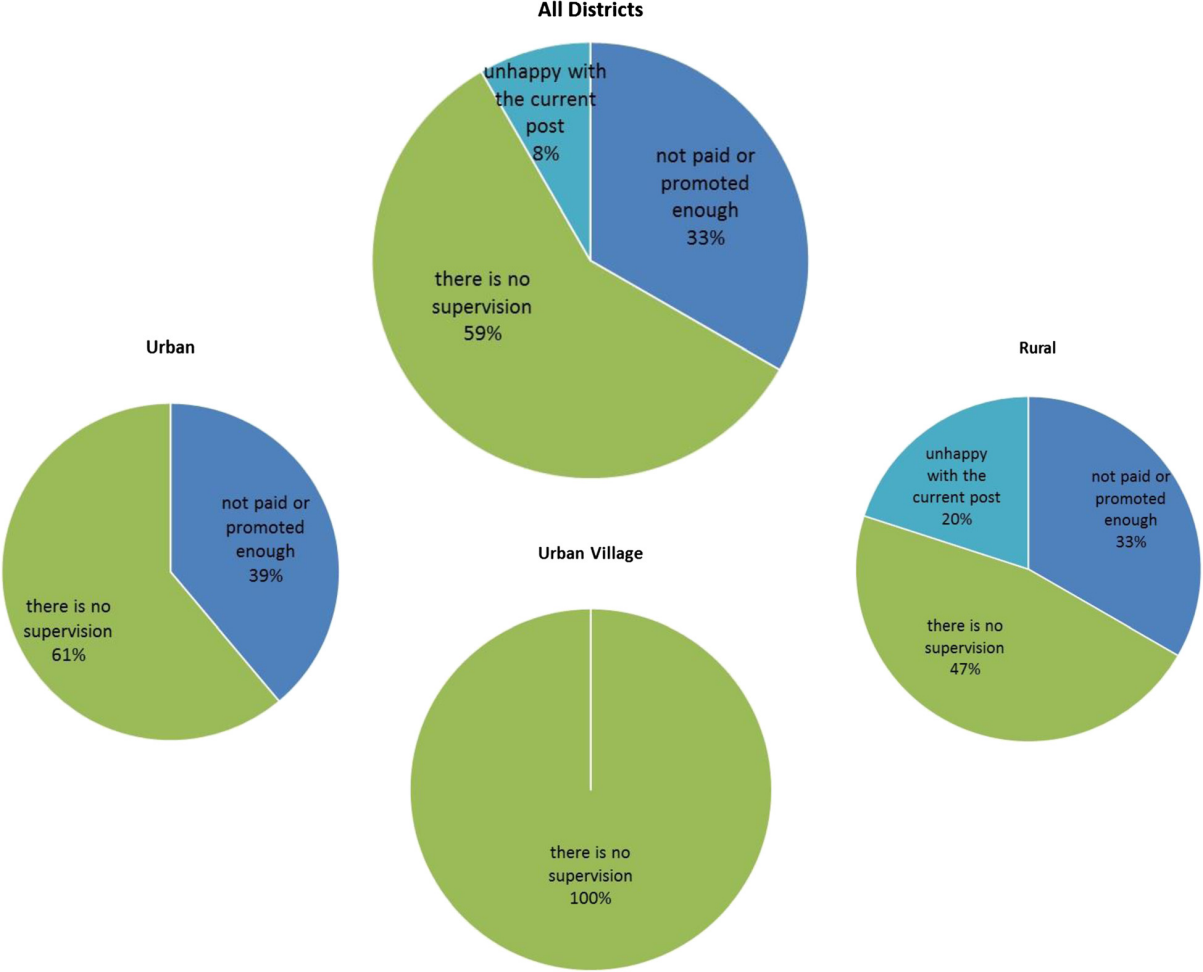
Theme “I am unsatisfied with my job.” This figure displays the frequency of sub-codes within the theme “I am unsatisfied with my job” emerging from interviews with providers in the study. The pie chart labeled *All Districts* presents the frequency of sub-codes in this theme in all the districts combined, while the three smaller pie charts show frequencies disaggregated by providers’ responses in urban, rural, and urban village districts included in the study

The categories and sub-categories under incentives were reviewed and further grouped into themes related to job dissatisfaction: (1) lack of promotions, (2) lack of supervision, (3) unclear future prospects, and (4) unhappiness with current post. Figure 1 displays the overall frequency and distribution of categories in this grouping or theme. Approximately half of the providers interviewed expressed dissatisfaction with their lack of progress and promotion, and a large proportion were dissatisfied with the lack of support and supervision. Unhappiness with current post was not a theme in urban and urban village districts although it was a dominant concern of providers in rural areas. The lack of supervision was the only theme expressed by providers in urban villages as affecting their job satisfaction.

The components of PITC were differentiated into how PITC was offered to patients, pre-test counseling, post-test counseling, and referral processes. In the PITC offer category, responses were included that related to the perspectives of providers on who and how the HIV test was offered to patients. Responses were grouped on why nurses do or do not offer and perform PITC, and which patients they referred to the lay counselors for the test. Less than a quarter of the nurses offered PITC to all patients. Some providers stated that they referred all patients they saw to the lay counselors; some referred only symptomatic patients, some referred only pregnant women, and some did not refer any patients because they felt patients were encouraged to test through health talks at the clinic or they already knew that they should get a test. Among all nurses making statements in relation to why they do not perform the test routinely, a large proportion stated they only perform the HIV test when the lay counselor is busy, felt strongly that it was the lay counselor’s job, and felt that it was too much work despite being trained to do it. Nurses in rural areas either never offered the test, which was likely related to the persistent lack of supplies in the area at the time of the study, or did it all of the time. In urban villages, nurses either never offered the test or did it sometimes to assist the lay counselor.

There were no distinct patterns that emerged on how providers delivered pre-test counseling. A quarter of the lay counselors interviewed mentioned they do sometimes use Ministry of Health provided flip charts to discuss HIV with patients during pre-test counseling. Others mentioned that they do not have time to pre-test counsel patients or that they relied on the health talks given at the clinic for that component of PITC.

Patterns did emerge in how post-test counseling was delivered by providers. Approximately half of the providers, and mainly the lay counselors, noted that post-test counseling of HIV-negative patients was not challenging and took less than 10 min. Close to half of the providers stated that they did not counsel HIV-negative patients because they felt patients already knew prevention messages.

In contrast to their views about HIV-negative patients, an overwhelming majority of the providers felt that post-test counseling of HIV-positive patients was challenging and took time. They felt that they did not have the skills to deal with positive test results, and faced challenges in dealing with men and patients with special needs. Providers who did not think post-test counseling of HIV-positive patients was challenging mentioned that patients were more accepting of results because they knew treatment was available or that they simply referred positive patients to an ART clinic for follow-up care. The patterns were the same for all three districts.

Responses related to the referral process were grouped according to whether they represented negative or positive views of the process. Approximately a third of providers felt that the referral process was good because they had treatment services onsite. The majority, however, expressed negative views related to patients not following recommendations, difficulty of monitoring patients that are HIV positive but not yet eligible for treatment, and not having treatment services onsite. It is to be noted that none of the providers’ responses explicitly mentioned follow-up care for HIV-negative patients. Similar patterns were seen in all three districts.

The majority of providers felt that space for HIV testing was inadequate pointing to issues in relation to the physical space such as the absence of a sink, window, and a fan in the HIV testing area. They consistently mentioned concerns with patients having to stand outside, patients seeing other patients, and patients—particularly men—not wanting to be seen outside of the HIV testing office. Those who expressed positive views of space said simply that it was working fine or patients had a seated and covered waiting area.

Finally, the majority of all providers interviewed expressed challenges with the supply of HIV rapid test kits. The analyses of this problem were only explored in terms of whether providers perceived it as a problem or not. All rural providers and the majority of urban providers identified the lack of supplies as a major barrier in delivering PITC. Providers in urban village sites did not raise supplies as an issue affecting their provision of PITC.

### Emerging themes

**Fig. 2:**
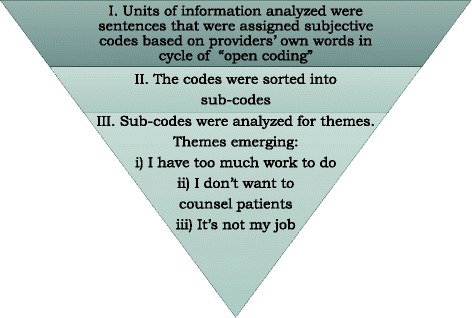
Inductive analysis schema. This figure presents the three cycles of coding used to inductively analyze provider interviews

The results from the first and second cycles of coding were analyzed further for emerging themes. Sub-codes were analyzed, and the researchers identified themes that they hypothesized were related to why providers were not implementing PITC. Figure 2 is a schema of the steps in inductive analysis from coding to thematic categorization. Table 2 shows the numbers and frequencies of codes grouped into the different themes. Three themes emerged that directly reflected provider attitudes towards PITC and why they did not perform it or found it challenging.

**Table 2:** Frequencies and distributions of the themes emerging from first and second cycle coding

Themes and patterns (higher-order codes)	Frequency of patterns
I have too much work to do	100 %
Overworked by patient scheduling issues	18 %
Overworked by staff scheduling issues	13 %
Overworked by systems issues	24 %
Overworked because of staff shortage	8 %
It is not my job	100 %
It is not a nurse’s job	100 %
I am unmotivated	100 %
Not paid or promoted enough	33 %
There is no supervision	58 %
Unhappy with the current post	8 %
I do not want to counsel patients	100 %
Counseling takes too much time	44 %
Counseling is difficult	56 %

### Theme 1: I have too much work to do

**Fig. 3:**
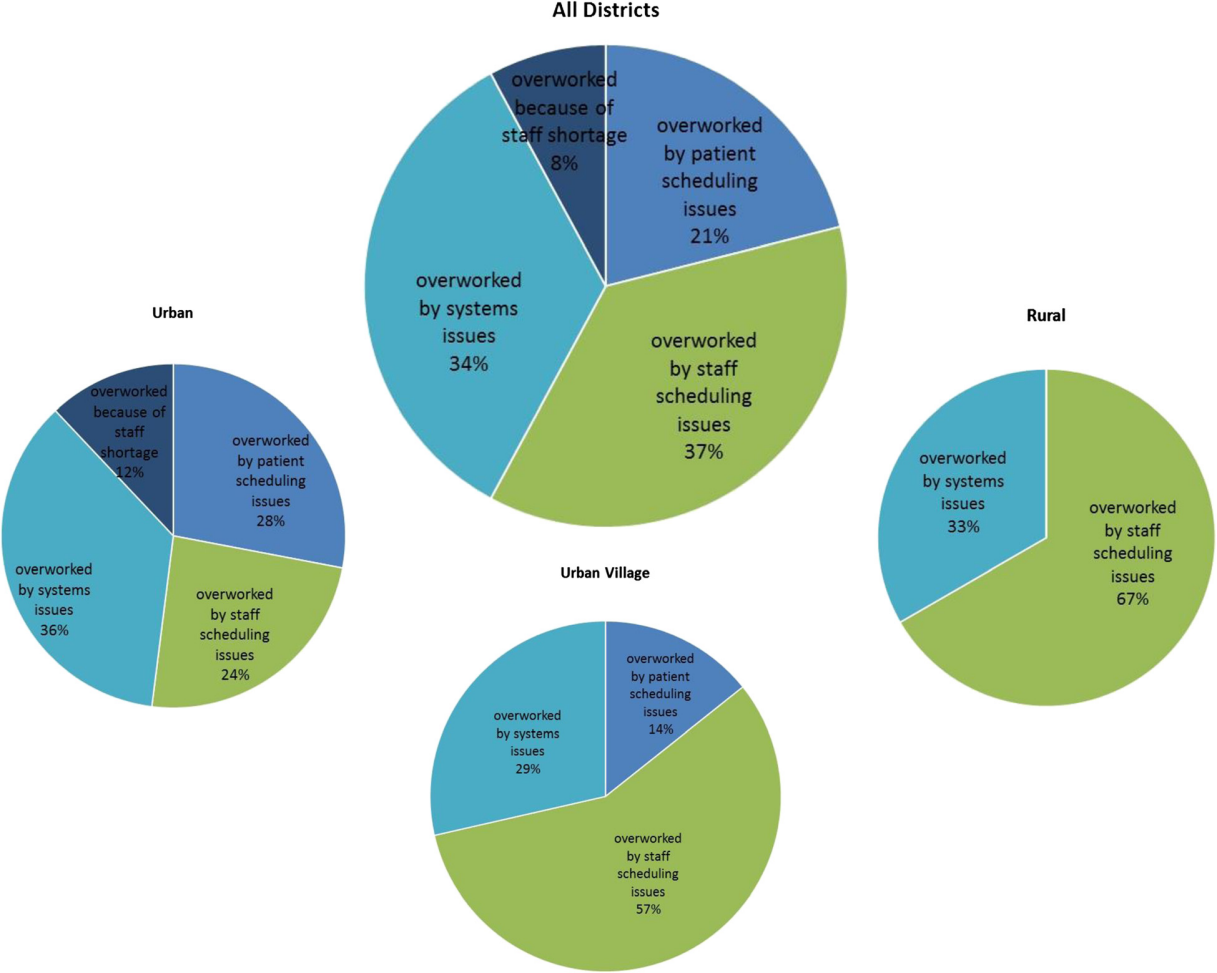
Theme 1 “I have too much work to do.” This figure displays the frequency of sub-codes within the theme “I have too much work to do” emerging from interviews with providers in the study. The pie chart labeled *All Districts* presents the frequency of sub-codes within this theme in all the districts combined, while the three smaller pie charts show frequencies disaggregated by providers’ responses in urban, rural, and urban village districts included in the study

Under the broad category of provider duties, the sub-codes with high density (mentioned three times or more by providers) were grouped into patterns. The categories all related to tasks that providers mentioned were reasons for their heavy workloads and were due to a shortage in staffing. These were grouped into the following: (1) workload challenges related to health system problems, e.g., categories of duties related to dispensing medication, dealing with lack of equipment, and transporting patients and blood samples; (2) workload challenges related to staff scheduling, e.g., being asked to shift from one task to the other, frustrations with unorganized schedules, and conducting home visits; (3) workload challenges related to patient scheduling, e.g., too many patients to be seen, managing queues, and irate patients; and (4) workload challenges related to shortage of staff, mainly a shortage in lay counselors. Figure 3 displays the overall frequency and distribution of categories in this theme and variations by district.

Among all the providers, the most dominant reason mentioned for high workload were challenges related to logistics and systems, followed by patient scheduling issues, staff scheduling issues, and staff. Patient scheduling and staff shortages disappeared as a pattern in rural areas, and staff shortages were not a pattern in urban villages. The following quotes exemplify the issues in this theme as raised by providers:

In addition to time, I think it’s the number of patients in the clinic, because we are really overwhelmed. The nurse patient ratio does not work in practice. That is one of the things that cause us to work in a hurry to complete the queue outside. And again, patients will be complaining outside. They will be complaining you are taking too much time with this patient, we have to go back to work, we are very sick. You know how they are. So it is the pressure from the patients and the fact that they are just too many patients per nurse.

Nurse #8, Gaborone District

A nurse is like, I don’t know, we will be giving health talks. Health talks should be given by a health educator, we are doing that. We are seeing general patients; taking specimens like lab techs; we are dispensing like pharmacy technicians; we are like doctors consulting. Everything. It is too much for us. For each unit, needs something to be written. Registers, registers, registers. We are writing.

Nurse #30, Kgalagadi South District

We are also short-staffed. Allocation is done in the morning and you can be doing something different every day.

Nurse #41, Mahalapye District

Ah, it is a lot. It is a lot because you take [the blood] and then it depends on communication. This other says no I’m not supposed to take blood there. As a driver you, ask him [to take the blood sample]. As a nurse, I can’t leave patients to drop off samples. So there used to be somebody that used to pick up blood sometimes. She doesn’t come, she doesn’t tell you I can’t come, so sometimes you throw, and sometimes you can force yourself to go.

Nurse #14, Gaborone District

[If other nurses assist with PITC] I will not be focusing on RHT and VCT. I am mostly focusing on PMTCT women and their children. So I think there is a lot of workload. I don’t have time for my women to go for some home visit…I don’t have time to check whether the program is doing right because of the workload.

Lay Counselor #39, Mahalapye District

### Theme 2: I do not want to do the counseling

**Fig. 4:**
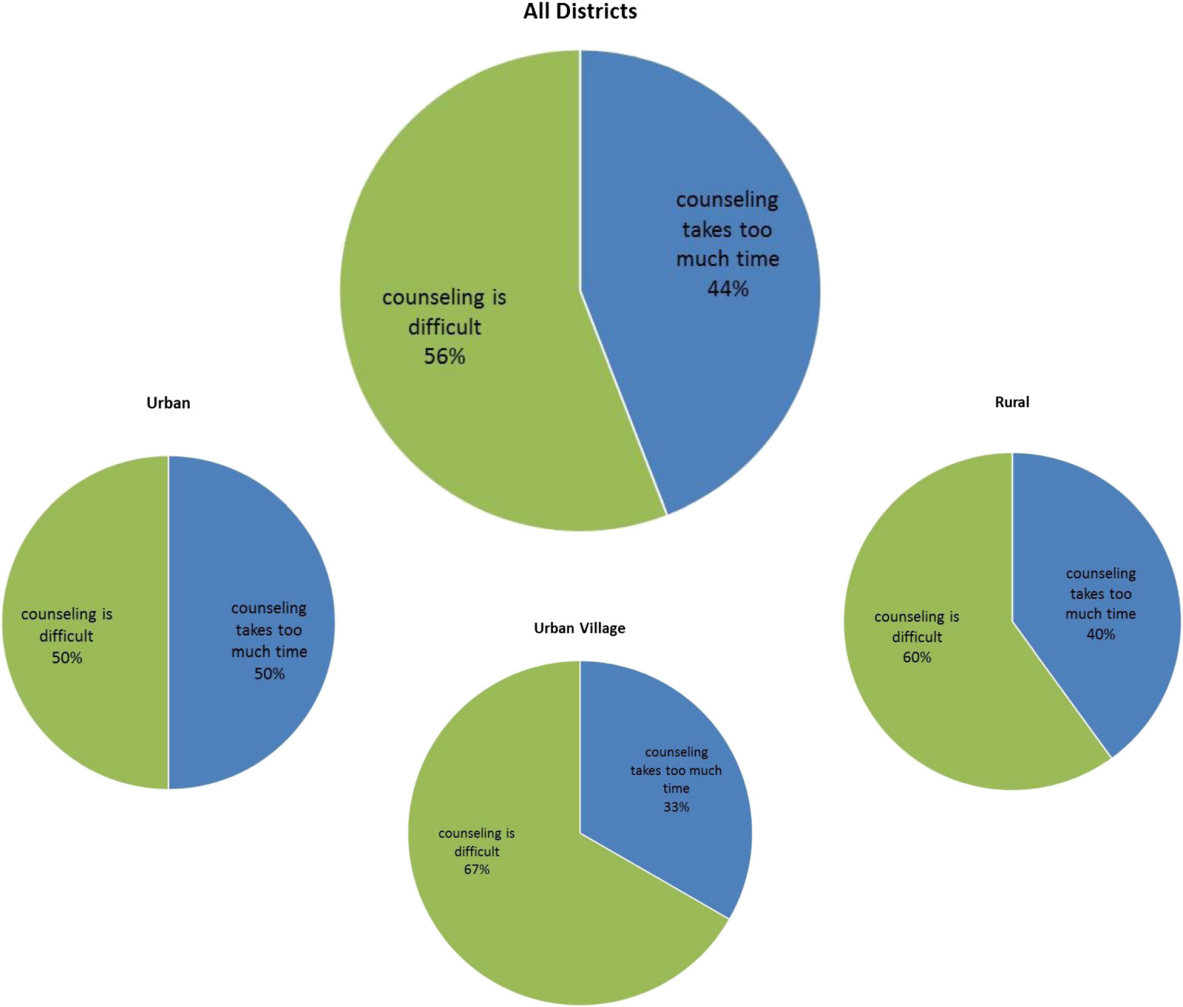
Theme 2 “I do not want to counsel patients.” This figure displays the frequency of sub-codes within the theme “I do not want to counsel patients” emerging from interviews with providers in the study. The pie chart labeled *All Districts* presents the frequency of sub-codes within this theme in all the districts combined, while the three smaller pie charts show frequencies disaggregated by providers’ responses in urban, rural, and urban village districts included in the study

Another theme that emerged from reviewing the sub-codes within and across different categories was that nurses either feared counseling patients or felt that counseling was too time consuming. These attitudes towards counseling led to their unwillingness to offer the test to patients. Figure 4 displays the overall frequency and distribution of categories in this theme and variations by district. Providers in rural areas and urban villages reported more often that counseling was challenging. The following quotes exemplify the issues in this theme as raised by providers:

When the patient comes in we begin with writing on the antenatal card. It takes a lot to write. Now you see the patient and now you won’t have the time to start counseling. It means when she is positive you have to sit with her for a longer time. Now, the other patients who come here early will go home late. That is how it is difficult for us. Unless, there is staff then we can do everything for the patient.

Nurse #11, Gaborone District

When the lay counselor is off it is challenging because post-test counseling…nowadays most people know about but still difficult to deal with a positive result.

Nurse #35, Mahalapye District

### Theme 3: It is not my job to perform PITC

Throughout the different categories and sub-codes, there emerged a pattern of nurses explicitly or implicitly expressing the view that PITC was not within a nurse’s job description or responsibilities. Some nurses explicitly made statements about themselves or nurses more broadly indicating that their training or job description did not include PITC. Others when asked about PITC immediately defined it as the lay counselors’ job. The following quotes exemplify the issues in this theme as raised by providers:

…doing everything even when there are things we are not supposed to do. We collect blood, we are not supposed to collect blood, we consult we are not supposed to consult, we issue drugs that side, we are not supposed to do that, there are people who have been trained to dispense drugs, we find ourselves forced to dispense drugs and we have to dispense them because if we are told to do something we just do it, so it frustrates us a lot.

Nurse #2, Gaborone District

Nurses say this is not for nurses, it is for lab technicians.

Nurse #42, Mahalapye District

Yes, RHT is offered here. There is a…now I can say there are lay counselors to do it. She is there…they are doing it in the caravan.

Nurse #22, Kgalagadi South District

### Resistance to performing PITC

**Fig. 5:**
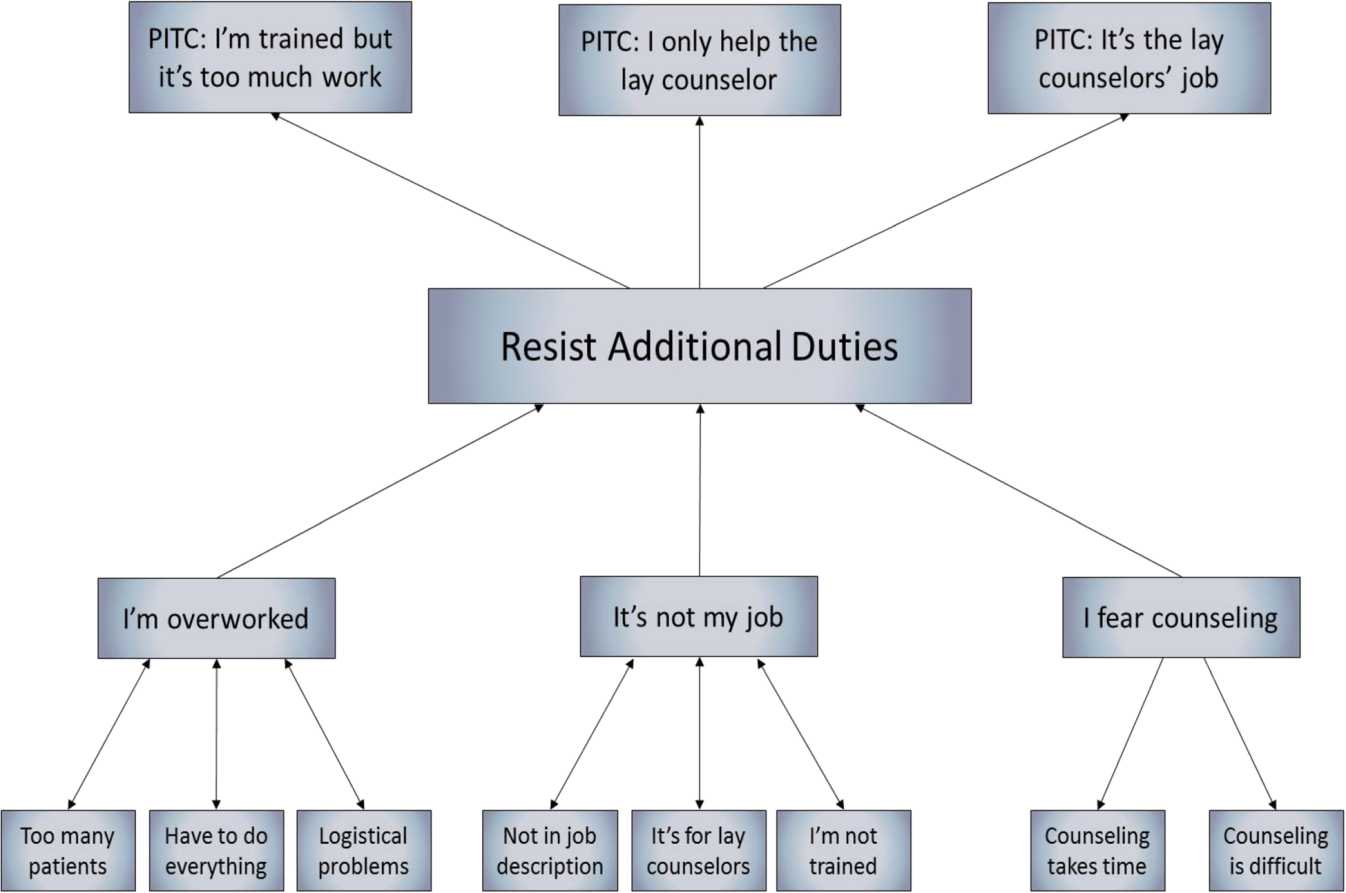
Matrix displaying the relationships between themes. This figure displays the matrix used to analyze how the themes emerging from the study lead to the hypotheses of why providers in the study resist delivering provider-initiated testing and counseling (PITC) in primary health care settings

The above three themes were further analyzed in relation to the responses that the providers gave in response to performing the different components of PITC. We constructed possible pathways that linked the themes to the provision of the different components of PITC. Figure 5 is the matrix depicting the pathways hypothesized to explain providers’ resistance to performing PITC or their inability to perform it as intended.

As one lay counselor puts it

Like being one in the caravan, I told her yesterday, I will be tired. I don’t have time to counsel. We just give someone the result and for her to go, you see. So we don’t have time to talk with the client to assist them in other ways. Because we will be looking at the line outside and we want to psh psh…(making sound of finishing up quickly).

Lay counselor #5, Gaborone District

## Discussion

The findings from this study showed that providers were resistant to take on the additional duties and tasks associated with PITC as part of their daily routines and standard of care. This resistance led to PITC not being offered at all or delivered in ways that compromise its quality and effectiveness. There were three themes that were identified as reasons for this resistance. First, providers stated that their workload made it difficult for them to offer PITC. Second, providers found counseling too time consuming or challenging. Third, nurses thought it was not their responsibility or within their job description to offer PITC. Each of these themes is discussed in more detail below.

The majority of providers, mainly those in urban facilities, found their workload impeding their ability to routinely offer PITC. While the providers themselves identified the shortage in staff as leading to their excessive workloads, our findings indicate that workload issues arose due to challenges with volume of patients seen, staffing issues, and logistical or system constraints that were related to having to transport patients and blood, lack of equipment, and procuring supplies. Alleviating these constraints may lead to better improvements in quality and productivity than simply adding more staff. A shortage in human resources is considered the main health systems constraint in scaling up HIV treatment programs [15, 16]. Human resource constraints have been central to the discussions on strengthening health systems with efforts focusing on addressing recruitment, skill mix, and distribution of providers [17, 18]. Another critical factor should be retaining and increasing the productivity of the workforce already in place [2]. A study in Tanzania to assess workforce productivity similarly concluded that sufficient gains in human resource supply can be made through improvements in staff productivity [19]. Quality improvement efforts at the facility level can also improve the supply of human resources [20]. For example, conducting quality improvement exercises to balance patient flow will free up staff time and improve their productivity. A review of procedures for blood collection and transport can identify systemic bottlenecks in the process that can be addressed at a facility, district, or national level. Thus, substantial gains in efficiency and improvements in quality can be attained by addressing the constraints that use up staff time and improve performance overall.

The issues of workload are particularly pronounced for lay counselors. Lay counselors in Botswana were originally hired to support nurses in prevention of mother-to-child transmission (PMTCT) delivery to provide continuous counseling for pregnant women, assist in dispensing infant formula, and other related tasks [21]. Tasks have been shifted to them gradually to perform the actual HIV test for pregnant women and to perform HIV testing and counseling for PITC and VCT patients. While lay counselors have undergone some training, albeit minimal, to take on these tasks, their pay and promotion scales and management and supervision structures have not been adjusted to reflect these additional responsibilities. In urban areas, we found that lay counselors were overwhelmed with the number of patients they have to see, and this was reflected in their inability to complete tasks related to PMTCT and PITC. In addition, even in areas in Botswana where workload was not of concern for lay counselors (rural and urban village sites), lay counselors felt under-valued and were dissatisfied with their promotion prospects and management support.

These findings are similar to the experiences of lay health workers in other countries in the region [22]. Task shifting should lead to the better use of currently existing human resources by diverting appropriate tasks to less specialized workers [17]. A few experiences with the task-shifting approach to service delivery suggest that it can lead to better access to care [18]. However, the impact on the quality of and access to health services from the perspective of providers has to be carefully considered in real settings. A recent review of the evidence on task shifting for maternal and reproductive health in low-income countries found that while sharing tasks may increase access for clients, health workers identified short-comings that included lack of training, supervision, career progression, and incentive packages [23].

Workload constraints aside, our findings identify a real shortage of lay counselors, particularly in urban settings, to meet the demands for HIV testing and counseling in facilities. A re-assessment is necessary to estimate the appropriate number of lay counselors required that factors in their expanded scope of work [24, 25]. This can be part of overall assessments done to estimate the human resource requirements in the country or a targeted assessment of service quantity, tasks, and productivity of lay counselors specifically. Modeling methods that incorporate a task-shifting framework and that have been used to assess other types of services could be explored to estimate and cost the human resource mix required to integrate PITC into primary health care [26].

The second theme was related to counseling and was raised by both nurses and lay counselors as a concern in delivering PITC. Interestingly, all providers perceived counseling of HIV negative patients as “easy” or not too time consuming. In regards to HIV-positive patients, some providers perceived their counseling to be adequate because they were able to refer patients to treatment. The majority who perceived it as challenging pointed to their lack of skill in dealing with patients with a positive result. While the content of counseling was not assessed in this study, these findings raise questions about the quality of counseling for this and other groups and the effectiveness of PITC as a prevention method. This indicates that along with efforts to address time constraints, which are also related to workload constraints, nurses and lay counselors should be better trained to counsel patients. Training is not only required to ensure counseling meets patients’ needs but also prepares providers with coping mechanisms to respond to the stress and burnout that occurs in interacting with HIV patients [27].

The third theme identified in the study was that nurses felt that PITC was not their responsibility or part of their job description. This view was expressed by nurses in all three districts. This could explain why in places where workload was not of concern and nurses were trained to perform PITC, nurses still referred patients to lay counselors. This view could reflect an expression of their professional identity in which they negatively perceive taking on PITC services specifically or HIV services more generally. Alternatively, it could be an outcome of other constraints they face such as workload, dissatisfaction with their job, or unwillingness to deal with HIV patients generally, which has been found to be the case in other studies in Sub-Saharan Africa [28, 29].

Another theme emerged in the analyses that could not be directly linked to PITC activities but could potentially influence the delivery of PITC was that nurses and lay counselors were unsatisfied with their jobs and unmotivated to work. The responses associated with this theme included complaints about lack of progress, low pay, lack of management support and supervision, and unhappiness with their current posts. There is some evidence that some of these aspects affect work performance [30]. One study in high-income countries found that a lack of organizational and managerial support for nursing “had a pronounced effect on dissatisfaction” and organizational and managerial support had a direct effect on nurse-assessed quality of care [31]. Another study in Malawi assessed factors that the researchers considered salient to job satisfaction and work performance such as levels of staffing and resources, management support, workplace relationships, and control over practice [32]. They found that these factors predicted job satisfaction and plans to leave their current position. Although the link between job satisfaction and work performance was assumed or conflated in the Malawi study, the complex relationship between job satisfaction and work performance cannot be ignored when considering the delivery of existing or new services.

It is important to underscore one of the patterns emerging in the job dissatisfaction theme—unhappiness with the current post, which was one of the dominant concerns in rural areas. There is a growing human resource management literature related to the factors of recruiting and retaining staff for remote and rural areas [33–35]. A review of the literature in middle- and low-income countries points to individual level, local and work-related, national, and international factors that influence attraction and retention of staff to rural areas [35]. Despite a lack of evidence on their effectiveness, there are strategies that have been proposed that involve recruitment and training targeted for rural practice, the use of incentives and compulsory services, and improving working and living conditions that should be considered in improving delivery of PITC in Botswana.

This study has qualitatively assessed providers’ attitudes and perspectives at the facility level that influence the delivery of PITC. The use of grounded-theory approach as an analytic method was suitable for this kind of exploratory and hypotheses building research. However, we make no claims of testing causal relationships between variables, and inferring effects and magnitudes from the results. The findings should be further replicated qualitatively and tested quantitatively in different contexts. The study also does not consider the connections between the facility level and the different levels of the health system. For example, supervision and management are very closely related to overall organizational aspects of the health system. Also, questions have to be answered about the level of control and autonomy facility-level providers have in managing work schedules and task-shifting responsibilities. Should this be done at the facility level or should this be regulated at a higher level? The methodology is, however, a useful one for implementation research and policy-making to better understand the supply of human resources and the barriers to delivering services in resource-constrained settings. The study does elucidate some of the issues that providers face at the facility level that influence PITC specifically and their work performance and ultimately health system outcomes in Botswana. Health systems strengthening efforts should be undertaken from the perspective of the providers taking into account their beliefs and perspectives in defining problems and their solutions [36]. The feasibility of integrating PITC into the standard provision of care and its acceptability for providers has been evaluated mostly under research or in small-scale settings [5, 37]. For PITC to succeed in real settings, the constraints that providers are required to work under must be addressed to improve existing programs.

## Conclusions

Using a grounded-theory approach to analyze data from qualitative interviews, we assessed why nurses in Botswana were or were not delivering PITC in a way that conformed to national plans and guidelines. The study found that nurses across facilities and districts were largely resistant to offering and delivering PITC for three reasons: they felt they were overworked and had no time to take on any additional duties; they felt that PITC was not their job but should be carried out by a different provider cadre, lay health workers; and they were afraid to counsel patients fearing the psychological distress of having to communicate a positive HIV test result. An important underlying theme was that both nurses and lay counselors were unsatisfied with pay and career prospects, which made them unmotivated to work in general. The distribution of these themes varied by urban and rural areas. Nurses in urban areas felt generally overworked and PITC was seen as contributing to the workload. While nurses in rural areas did not feel overworked, they felt that PITC was not their job and were generally unmotivated because of unhappiness with their posts. These findings show that the attitudes of providers and the barriers they faced played a critical role in whether and how PITC was being implemented. Provider factors should be considered in the improvement of existing PITC programs and the design of new ones.

## Abbreviations

HTC: HIV testing and counseling
PITC: provider-initiated testing and counseling for HIV
PMTCT: prevention of mother-to-child transmission
VCT: voluntary counseling and testing
